# Heart Rate Variability in Patients with Hypertension: the Effect of Metabolic Syndrome and Antihypertensive Treatment

**DOI:** 10.1155/2020/8563135

**Published:** 2020-10-13

**Authors:** Małgorzata Maciorowska, Paweł Krzesiński, Robert Wierzbowski, Grzegorz Gielerak

**Affiliations:** Department of Cardiology and Internal Diseases, Military Institute of Medicine, Szaserow Street 128, 04-141 Warsaw 44, Poland

## Abstract

Metabolic syndrome (MetS) is a combination of factors which, collectively, increase cardiovascular risk to a greater extent than each of them separately. Previous studies showed high cardiovascular risk to be associated with autonomic nervous system dysfunction. The purpose of this study was to assess the effects of antihypertensive treatment on heart rate variability (HRV) in patients with hypertension (HTN), depending on cooccurrence of MetS. 118 patients with uncontrolled HTN were enrolled to the study. HRV was compared among patients with and without MetS (MetS [+], *n* = 70) at baseline and following 12 months antihypertensive treatment. The HRV indices measured from RR intervals recorded form using 24-hour ambulatory electrocardiography. The measured HRV domains were the standard deviation of the average of NN intervals [SDNN], square root of the mean of the sum of the squares of differences between adjacent NN intervals [rMSSD], percentage of NN50 [pNN50], low frequency [LF], high frequency [HF], total power of variance of all NN intervals [TP], and LF/HF ratio. Baseline parameters: SDNN, rMSSD, pNN50, and HF were significantly lower in the MetS[+] compared to the MetS[-] subgroup (*p* < 0.05). After a 12-month antihypertensive treatment, MetS[+] patients achieved a significant improvement in parameters: SDNN, rMSSD, pNN50, and TP (*p* < 0.05), while the changes in HRV observed in the MetS[-] subgroup were not statistically significant. The cooccurrence of HTN and other components of MetS is associated with disturbances of the autonomic balance. HTN control has a beneficial effect on HRV, with the effect being more evident in patients with MetS.

## 1. Introduction

Metabolic syndrome (MetS) is a combination of cardiovascular risk factors, such as abdominal obesity, hypertension (HTN), abnormal glucose metabolism, and atherogenic dyslipidemia (hypertriglyceridemia and low HDL levels) [1]. Combined, these factors were shown not only to adversely affect cardiovascular hemodynamics [2] but, above all, to increase cardiovascular risk to a greater extent than each of them individually [3–5].

MetS is very common in general population, but the prevalence is highly influenced by different diagnostic criteria used. According to the 2003-2012 data from NHANES prevalence of the MetS in the United States was 33%, with prevalence growing with age, 18.3% among those 20-39 years to 46.7% in those aged 60 years or older [6], the survey conducted in the Chinese elderly population showed increase prevalence of MetS from 50.4% in 2001 to 58.1% in 2010 [7]. European MetS prevalence, using the International Diabetes Federation diagnostic criteria, has been estimated as 41% in men and 38% in women [8]. Previous studies show that MetS is a significant predictor of cardiovascular morbidity and mortality [3, 4, 8]. Mediterranean Hypertensive Population patients with three or more components of MetS had threefold higher risk for cardiac events, 2.59 for cerebrovascular, and 2.26 for total cardiovascular events compared with those with no other component [9]. Consequently, the total cost of the complications of the syndrome including the cost of health care and loss of potential economic activity is huge.

Earlier studies demonstrated the complex pathophysiology of MetS by identifying the role of genetic and environmental factors, insulin resistance, inflammation, and oxidative stress [10, 11]. Therefore, MetS is unequivocally a systemic condition. There are a number of papers showing MetS-associated autonomic nervous system (ANS) imbalance, manifesting as elevated sympathetic and diminished parasympathetic activity. This phenomenon has been observed for all MetS components, including HTN [12–14]. The sympathetic hyperexcitability appears to have primarily consequences for the development of obesity and insulin resistance as well as hypertension, what is connected with elevated urinary and plasma noradrenaline levels, TNF*α* contribution, elevation of adipokine levels, renal upregulation of glucose transporters, *β*-adrenoceptor sensitization, and angiotensin II release [15].

There are many methods that allow both direct and indirect assessment of the ANS function. An indirect method, relatively easily accessible, is evaluation of heart rate variability (HRV) in ambulatory electrocardiography. It has been postulated that a noninvasive assessment of ANS activity, e.g., via analyzing heart rate variability (HRV), may be useful in identifying patients at risk of developing MetS in the future [16]. Particularly, 24-hour recordings seem to be more reliable to clarify to what extent HRV is altered in MetS. HRV is frequently abnormal in patients with clinically overt cardiovascular conditions, such as coronary artery disease and heart failure [17, 18], strongly related to poorly controlled cardiometabolic risk factors.

There are studies which have examined the association between metabolic syndrome and heart variability, but fewer take the challenge to evaluate the effectiveness of applied treatment in primarily not treated hypertensives. Therefore, the purpose of this study was the assumption that in the case of patients with HTN and MetS, it seems of clinical importance to determine how much the concomitant metabolic disturbances affect HRV and whether or not antihypertensive treatment modifies HRV to the same extent as in hypertensive patients without MetS.

## 2. Methods

### 2.1. Study Population

This study analyzed the data collected from patients recruited for the FINEPATH research study (ClinicalTrials.gov Identifier NCT01996085), which had been conducted at the Department of Cardiology and Internal Diseases of Military Institute of Medicine, in the period 2011–2014. The FINEPATH study enrolled 144 patients with uncontrolled HTN, defined as elevated blood pressure (≧140/90 mmHg) for at least 3 months prior to study enrollment, without pharmacotherapy at baseline. The key patient characteristics were presented in one of the earlier publications by our team [19]. The FINEPATH study was a prospective, randomized, controlled study to assess a novel HTN treatment. The following drug classes were used after baseline assessment: beta-blockers, angiotensin converting enzyme (ACE) inhibitors, angiotensin receptor blockers (ARB), calcium channel blockers (CCB), and diuretics, either alone or in combination. The final follow-up visit was conducted 12 months after treatment initiation. The exclusion criteria were (1) secondary HTN; (2) chronic kidney disease (glomerular filtration rate (GFR) < 60 mL/min/1.73m^2^ calculated by the MDRD formula); (3) other severe comorbidities, including systolic heart failure, cardiomyopathy, significant arrhythmias, significant valvular heart disease, chronic obstructive pulmonary disease, previously diagnosed diabetes mellitus, polyneuropathy, and peripheral vascular disease; (4) age < 18 years and > 75 years; (5) body mass index (BMI) > 40 kg/m^2^; (6) psychiatric disorders precluding the patient's cooperation; (7) any nonsinus hearth rhythm (including permanent cardiac pacing); and (8) ECG tracings containing > 300 premature complexes and artifacts. The study protocol had been approved by the Institutional Review Board at Military Institute of Medicine (Approval No. 21/WIM/2011), and each patient had provided his or her written consent.

### 2.2. History and Physical Examination

History-taking and physical examination focused particularly on cardiovascular risk factors: age, sex, office systolic blood pressure (SBP), office diastolic blood pressure (DBP), smoking, family history of heart disease, and BMI. The following parameters were measured in each patient: fasting blood glucose (mg/dL), creatinine (mg/dL), high-density lipoprotein (HDL) cholesterol (mg/dL), low-density lipoprotein (LDL) cholesterol (mg/dL), triglyceride levels (mg/dL), and estimated GFR (MDRD eGFR) (mL/min/1.73m^2^). Metabolic syndrome was diagnosed based on the International Diabetes Federation (IDF) criteria [20]: central obesity—waist circumference > 94 cm for European men and >80 cm for European women plus any two of the following four factors: triglyceride levels ≥ 150 mg/dL (≥1.7 mmol/L), or treatment for hypertriglyceridemia; HDL − cholesterol levels < 40 mg/dL (<1.03 mmol/L) in men or <50 mg/dL (<1.29 mmol/L) in women, or treatment for low HDL; SBP ≥ 130 mmHg or DBP ≥ 85 mmHg, or treatment for previously diagnosed HTN; fasting plasma glucose ≥ 100 mg/dL (≥5.6 mmol/l), or previously diagnosed diabetes mellitus.

### 2.3. 24-Hour Ambulatory Electrocardiography (Holter Monitoring)

All patients underwent 24-hour ambulatory electrocardiography with 3-channel digital LifeCard CF recorders (Del Mar Reynolds Medical - Spacelabs Healthcare; US) to assess any arrhythmias, minimum, mean, maximum heart rate (HR), and HRV.

The time-domain and spectral (frequency-domain) HRV parameters were analyzed with the use of the Impresario Symphony Holter Analyzer system (Del Mar Reynolds Medical, Spacelabs Healthcare Ltd/UK). The preliminary processing of the obtained ECG tracings included a review and correction of wrongly classified beats, artifact elimination, and evaluation of any arrhythmias and ST-segment changes. Only the R-R intervals between normal QRS complexes were analyzed, with the R-R intervals preceding and following ventricular premature complexes excluded from analysis. The author who analyzed the HRV data was blinded to which patients had metabolic syndrome and which patients did not.

The patients were asked to avoid intense physical activity, smoking, and drinking alcohol. They were recommended to stop their activity at 10 p.m. and sleep till 6 a.m. The examinations were performed in hospital settings that limited the influences of other confounding factors, such as diet and work stress.

### 2.4. Analysis of HRV Time-Domain Parameters

The automatically detected time-domain parameters included in our analysis were daytime (*parameter*_day), nighttime (*parameter*_night), and 24-hour (*parameter*_24h) HRV parameters. The time-domain analysis of HRV provides mainly quantitative data, illustrating the extent of variability. The following parameters were used in our comprehensive HRV assessment: the standard deviation of the average of NN intervals in milliseconds (SDNN)and - for assessing the parasympathetic component in the area under the curve - the squares for assessing the parasympathetic component in the area under the curve—, the square root of the mean of the sum of the squares of differences between adjacent NN intervals in milliseconds (rMSSD), and the percentage of NN50 (pNN50) [21].

### 2.5. Analysis of Frequency-Domain HRV Parameters

The analysis of frequency-domain parameters was conducted with fast Fourier transform (FFT). Out of the total recorded spectrum, our analysis included the normalized low-frequency (LF) (0.05–0.15 Hz) and high-frequency (HF) (0.15–0.4 Hz) values, LF/HF ratio, and total power of variance of all NN intervals [TP]). A spectral analysis was conducted for each hour out of the 24-hour period. Subsequently, the mean daytime and nighttime values were calculated and the day/night ratio. The HF parameter was considered to be an indicator of the parasympathetic activity. LF values depend on the effect of both the vagus nerve and sympathetic tone. At rest, LF shows a combined effect of the sympathetic and parasympathetic nervous systems, whereas following sympathetic stimulation (e.g., standing up, exercise, and psycho-emotional stress), LF reflects mainly the activity of the sympathetic nervous system. The relationship between LF and HF (LF/HF ratio) reflects the sympathetic-parasympathetic balance [21].

### 2.6. Statistical Analysis

Statistical analyses were conducted with Statistica 12.0 (StatSoft Inc.). The distribution and normality of data were assessed visually and with the Kolmogorov-Smirnov test. Continuous variables were presented as the mean ± standard deviation (SD), whereas categorical variables were presented as absolute and relative values (percentages). A comparison analysis was conducted for two subgroups: MetS[+] (patients with other MetS factors apart from HTN) and MetS[-] (patients not diagnosed with MetS). Student's *t*-test was used for normally distributed data, whereas the Mann-Whiney U-test was used for the data with nonnormal distribution. Spearman's rank correlation coefficient was performed to investigate the relations between changes in BP and HRV parameters. The assessment of treatment effects for subgroups separately involved the use of the Wilcoxon signed-rank test. And the nonparametric Friedman test as an alternative to the two-way repeated measures ANOVA was performed in order to determine whether there is a significant interaction between MetS and effect of time (treatment). The *p* value of <0.05 was considered statistically significant.

## 3. Results

**Figure 1:**
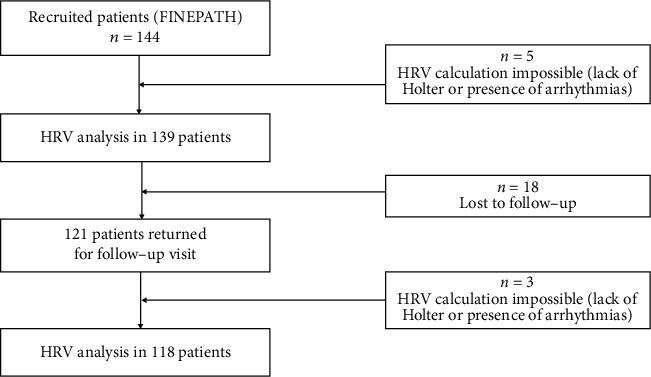
Study patient flow chart.

Out of the 144 patients included in the FINEPATH study, 139 underwent Holter monitoring with HRV analysis, with data from 118 patients (who returned for the final follow-up visit) included in the final analysis. Eighteen patients had been lost to follow-up (they failed to return for the visit after 12 months), and the HRV of 3 patients could not be calculated due to a lack of Holter monitoring or the presence or arrhythmias) (Figure 1).

### 3.1. Baseline Characteristics

The study group comprised mostly males (69%). The mean age was 46 years, mean HR 74 bpm, and mean blood pressure 141/90 mmHg. All patients enrolled to the study were Caucasian. More than half (59%) of the patients met the MetS criteria (Table 1). Those subjects were slightly (borderline *p*) and more frequently males.

### 3.2. Comparison of Baseline HRV Values

Table 2 presents HRV parameters in patients stratified by the presence or absence of MetS prior to antihypertensive treatment initiation. In comparison with the MetS[-] subgroup, the patients from the MetS[+] subgroup of comparable age, HR, and blood pressure values showed significantly lower values of the following time-domain HRV parameters: SDNN_24h (*p* = 0.048), SDNN_day (*p* = 0.015), rMSSD_24h (*p* = 0.002), rMSSD_day (*p* = 0.002), rMSSD_night (*p* = 0.020), pNN50_24h (*p* = 0.0008), pNN50_day (*p* = 0.0004), pNN50_night (*p* = 0.018), and the spectral parameter HF_night (*p* = 0.041).

### 3.3. Assessment of 12-Month Treatment Effects

After 12-months of antihypertensive treatment, there was significant reduction in blood pressure both in the MetS[+] and MetS[-] subgroups, down to 120.1/77.2 mmHg (*p* < 0.001) and 121.2/77.6 mmHg (*p* < 0.001), respectively. A similar effect was also observed in terms of HR, with HR of 69.1 bpm (*p* < 0.001) and 66.5 bpm (*p* < 0.001), respectively. The BP control (<140/90 mmHg) was achieved in 68 (97%) pts with MetS and 42 (88%) pts without MetS (*p* = 0.041). The 12-month follow-up showed no changes in body weight either in the MetS[+] (91.1 ± 13.5 kg before vs. 91.3 ± 13.4 kg after treatment; *p* = 0.73) or in the MetS[-] subgroup (87.0 ± 15.9 kg before vs. 86.6 ± 15.3 kg after treatment; *p* = 0.84).

Antihypertensive monotherapy was used in 47.1% of patients (a CCB in 0.8%, beta-blocker in 5.0%, ARB in 4.2%, diuretic in 3.4%, ACE inhibitor in 33.6%). Combination antihypertensive therapy was used in 50.4% of patients, with two-drug combination regimens of ACE inhibitor plus diuretic in 16.8% of patients, ACE inhibitor plus beta blocker in 10.9%, ACE inhibitor plus CCB in 7.6%, ARB plus diuretic in 3.4%, ARB plus beta-blocker in 0.8%, and ARB plus CCB in 0.8%. Three-drug combination regimens, used in 7.5% of patients, included ACE inhibitor plus beta-blocker plus CCB in 2.5% of patients, ARB plus CCB plus diuretic in 0.8%, ACE inhibitor plus beta-blocker plus diuretic in 2.5%, and CCB plus beta-blocker plus diuretic in 1.7%. No antihypertensive medications were used in 2.3% of patients, who only received nonpharmacological recommendations. Statins were introduced in 16 patients with MetS (22.9%).

**Figure 2:**
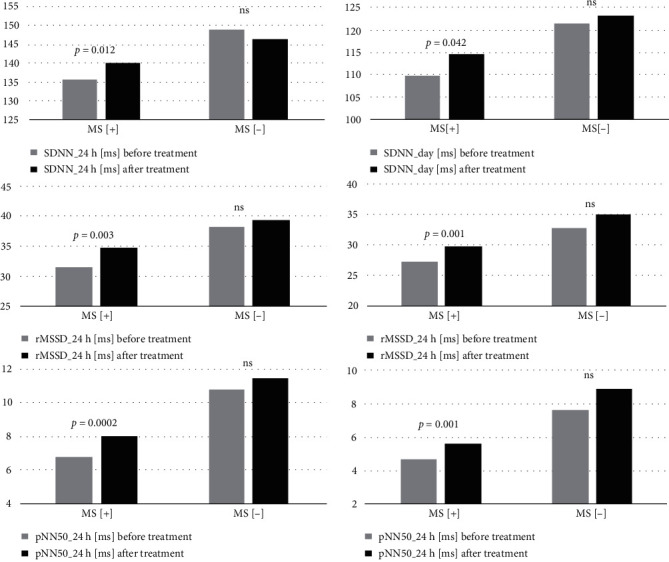
Comparison of selected HRV parameters before and after 12-month treatment, in patients with HTN stratified by concomitant MetS.

Table 3 presents a comparison of HRV parameters in patients with HTN stratified by the presence or absence of concomitant MetS at 12 months of antihypertensive treatment. MetS[+] patients achieved a significant improvement in their HRV as shown by time-domain parameters: SDNN_24h (*p* = 0.012), SDNN_day (*p* = 0.042), rMSSD_24h (*p* = 0.003), rMSSD_day (*p* = 0.001), rMSSD_night (*p* = 0.042), pNN50_24h (*p* = 0.0002), pNN50_day (*p* = 0.001), and the frequency-domain parameter of TP_day (*p* = 0.026). The results achieved in the MetS[-] subgroup also suggest a favorable effect of treatment; however, the observed differences did not reach the adopted level of significance and were lower than in the MetS[+] subgroup also in terms of absolute values (Table 3 and Figure 2). Friedman's test revealed the significant interaction between MetS and effect of treatment for SDNN_day, rMSSD_day, pNN50_24h, pNN50_day, and TP_day. The significant correlations were observed for 12-month changes in diastolic blood pressure and some HRV parameters (SDNN, rMSDD, and pNN50) in both MetS[+] and MetS[-]. The effect on systolic blood pressure was less related to HRV [Supplementary Table 1].

## 4. Discussion

Our findings indicate a considerable effect of metabolic disorders on the HRV in patients with HTN. Implemented hypertensive therapy was effective in both subgroups, but the MetS patients were those who seem to benefit more from the treatment with respect to sympatovagal balance.

The baseline values of HRV parameters obtained in our study and the impact of MetS are consistent with the data reported in the available literature [12–14, 22–28]. A 2013 study by Li et al., which aimed to assess the relationship between MetS severity and ANS function, demonstrated independent negative correlations of two MetS components (fasting plasma glucose and HTN) with ANS function [23]. Moreover, an earlier study (Twins Heart Study) conducted in 288 pairs of twins showed a relationship between MetS and decreased HRV parameters, both in individual analyses and in the analyses of the twin pairs. Additionally, HRV parameters were found to be decreased in individuals with more MetS components [24]. American researchers [25] reached a similar conclusion while assessing the HR and HRV parameters in patients stratified by their fasting glucose (FG) levels and other concomitant MetS components. This American study demonstrated lowering of most of the evaluated HRV parameters (particularly the SDNN, standard deviation of the 5-minute average NN intervals [SDANN], TP, ultra-low frequency [ULF], and very low frequency [VLF] power) in patients with markedly elevated FG (6.1–6.9 mmol/L) and type 2 diabetes (with FG > 6.9 mmol/L or on antidiabetic medication or insulin) in comparison with the patients with normal (4.5–5.5 mmol/L) and slightly elevated (5.6–6.0 mmol/L) FG. The patients with normal to slightly elevated FG who met more than 2 MetS criteria showed decreased HRV (SDNN, SDANN, TP, and ULF) in comparison with the patients meeting at most one MetS criterion. In patients with diabetes or markedly elevated FG, MetS was associated with decreased HRV compared with the HRV in patients without MetS. These American findings were consistent with those of the Finnish authors whose 1998 study demonstrated significantly decreased HRV parameters (SDANN, TP, VLF, LF) in hypertensive patients with insulin resistance in comparison with both hypertensive patients without insulin resistance and normotensive patients. The HF parameter (*p* < 0.001) and baroreflex sensitivity (*p* < 0.05) were diminished in both hypertensive groups [28]. A prospective study by Balcioğlu et al. [29] (*n* = 240) showed significantly decreased HRV parameters (SDNN, SDNN index, SDANN, rMSSD, pNN50) in 24-hour Holter recordings in comparison with those in the control group. Unlike in the studies mentioned above, the lowering of HRV parameters correlated only with fasting glucose levels, with no differences between the groups in terms of the remaining 4 diagnostic criteria of MetS (notably, both study groups included patients with HTN).

Our study demonstrated the effects of antihypertensive treatment on HRV parameters to be beneficial, particularly in the group of patients with MetS. The more altered HRV at baseline may partly explain greater reduction after 12 months of treatment in MetS. No other mechanism can be identified basing on our data. Our findings are consistent with earlier reports indicating beneficial effects of antihypertensive treatment on HRV. However, there is no clear consensus which hypotensive drugs are the most beneficial in terms of the sympatovagal balance. Some earlier studies demonstrated beta-blockers and ARB to be particularly beneficial in that respect [30–32]. Moreover, some other reports indicated that ARB treatment yielded better effects than treatment with ACE inhibitors and beta-blockers [33–35]. One prospective, randomized Japanese study compared the effects of ARB treatment in MetS patients randomized into three therapeutic groups (telmisartan, candesartan, diet therapy) [35]. At 6 months, the study showed a comparable lowering of blood pressure in both drug-treated groups. However, ARB treatment yielded increased baroreflex sensitivity, increased high-molecular-weight adiponectin levels, and improved endothelial dysfunction (in this last respect, a more pronounced effect was achieved with telmisartan). Moreover, the telmisartan group showed significantly decreased norepinephrine levels, blood pressure variability, and the spectral HRV parameter of the LF/HF ratio (*p* < 0.05). A study by Menzes et al. showed improvement in all HRV parameters (SDNN, pNN50, LF; *p* < 0.001) following a 3-month treatment with an ACE inhibitor (enalapril or ramipril) in contrast with the control group [31]. Petretta et al. assessed the effect of a 12-month lisinopril treatment on HRV and, for the entire study population, observed an increase only in the nighttime HF parameter in comparison with baseline values [33]. However, the subgroup of patients with left ventricular mass normalization showed increased both daytime and nighttime HF, as well as increased nighttime TP and VLF. A 2010 study by Pavithran et al. examined 150 patients newly diagnosed with HTN, divided into five 30 patient groups, each receiving one of the following: amlodipine, atenolol, enalapril, hydrochlorothiazide, or an amlodipine+atenolol combination. Only the amlodipine+atenolol group showed a significant change in HRV (increased total variability of RR intervals and HF spectral power) [36]. There were also studies attempting to evaluate the effect of individual CCB medicines on HRV parameters [37–40]. The available data on the effect of amlodipine on the HRV are contradictory. Individual authors report either an insignificant-to-absent effect of this drug on the ANS activity, or enhanced sympathetic activity, or—conversely—vagus nerve stimulation [37–39]. A prospective, randomized study by Karas et al. (*n* = 57) evaluated the effects of treatment with amlodypine, ramipril, and telmistartan on HRV spectral analysis and plasma norepinephrine and epinephrine level measurements [41]. Following amlodipine treatment, an increased daytime sympathetic activity and decreased nighttime parasympathetic activity, together with increased plasma norepinephrine levels, were observed. Telmisartan treatment yielded considerably increased parasympathetic activity without changes in plasma norepinephrine levels, whereas ramipril increased the parasympathetic activity only during the day.

For both baseline comparison and treatment effects, time-domain HRV parameters revealed to better diversify the presence of metabolic burden than frequency-domain HRV parameters. The frequency-domain HF power is assumed to correspond to the frequency of breathing, reflecting respiratory sinus arrhythmia. More controversies concern LF power. Some authors undermine that LF power is and index of cardiac sympathetic tone and are even more willing to claim that it reflects baroreflexes [42].

Considering high prevalence of MetS around the world, our finding may concern a wide range of patients. Blood pressure control seems to complement the intervention based on diet and physical activity to reverse MetS and prevent cardiac autonomic neuropathy [43].

### 4.1. Strengths and Limitations

The strength of our study is the enrollment of hypertensive subjects, some of them with MetS, but no significant comorbidities. Moreover, there were no bias of previous hypotensive treatment at baseline assessment. Some limitations should be also considered. One is the small size of the study population and thus a small size of individual subgroups. Therefore, our analyses may be underpowered, and the findings need to be confirmed in a larger study group. Another limitation is the difference in sex proportions between MetS subgroups that could bias baseline comparison. Moreover, we would like to point out that the absence of any monitoring in terms of pharmacotherapy, other than the patients' antihypertensive treatment report, may have affected our results. Our study assessed neither patients' physical activity nor the effect of treatment on the metabolic dysfunction. In terms of Discussion, we would like to emphasize the issues with comparing individual studies due to the differences in study protocols, the adopted diagnostic criteria of MetS, and the length of analyzed electrocardiographic recordings. Moreover, only a handful of studies included separate groups of patients with uncomplicated HTN and those with HTN and concomitant metabolic disorders.

## 5. Conclusions

Our findings confirm that the cooccurrence of HTN and other components of MetS is associated with differences in HRV and its modulation by hypotensive medicines. Blood pressure control has a beneficial effect on HRV, with the effect being more evident in patients with MetS.

## Figures and Tables

**Table 1:** Baseline patient characteristics (the entire study population).

	All [*n* = 118]	MetS [+], *n* = 70	MetS [-], *n* = 40	*p*
Age [years]	46 ± 10	48 ± 10	44 ± 11	0.054
Males	80 (68)	55 (79)	25 (52)	0.002
HR [bpm]	74 ± 11	74 ± 10	73 ± 12	0.382
OSBP [mmHg]	141 ± 13	142 ± 11	140 ± 15	0.284
ODBP [mmHg]	90 ± 9	90 ± 9	89 ± 10	0.500
MetS (IDF)	70 (59)	70 (100)	0 (0)	—
Creatinine [mg/dL]	0.83 ± 0.16	0.85 ± 0.14	0.82 ± 0.19	0.342
eGFR [mL/min/1.73m^2^]	99 ± 17	99 ± 16	99 ± 19	0.683
Glucose [mg/dL]	99 ± 12	103 ± 12	93 ± 7.2	<0.001
Total cholesterol [mg/dL]	225 ± 39	226 ± 40	222 ± 37	0.828
HDL [mg/dL]	59 ± 18	52 ± 15	68 ± 19	<0.001
LDL [mg/dL]	144 ± 34	148 ± 31	139 ± 39	0.294
TG [mg/dL]	152 ± 76	186 ± 73	103 ± 48	<0.001
BMI [kg/m^2^]	29 ± 4	30 ± 4	27 ± 4	<0.001
Smokers	23 (19)	10 (21)	13 (19)	0.761
Statin	3 (2.5)	3 (4)	0 (0)	0.146

Data presented as mean ± SD/*n* (%). BM: body mass index; eGFR: estimated glomerular filtration rate; HR: heart rate; IDF: International Diabetes Foundation criteria; MetS: metabolic syndrome; ODBP: office diastolic blood pressure; OSBP: office systolic blood pressure; TG: triglycerides.

**Table 2:** Comparison of HRV parameters in subgroups stratified by concomitant MetS (before treatment initiation).

	MetS [+], *n* = 70 Mean ± SD	MetS [-], *n* = 48 Mean ± SD	*p* value
SDNN_24h [ms]	135.5	149.1	0.048
SDNN_day [ms]	109.8	121.5	0.015
SDNN_night [ms]	90.5	94.6	0.473
rMSSD_24h [ms]	31.5	38.2	0.002
rMSSD_day [ms]	27.3	32.7	0.002
rMSSD_night [ms]	40.0	49.0	0.020
pNN50_24h [%]	6.80	10.79	0.0008
pNN50_day [%]	4.66	7.63	0.0004
pNN50_night [%]	12.65	19.28	0.018
LF/HF_day [-]	4.50	3.88	0.625
LF/HF_night [-]	3.04	2.01	0.048
LF_day [n.u.]	70.5	70.0	0.939
LF_night [n.u.]	62.3	55.8	0.061
HF_day [n.u.]	23.0	24.1	0.524
HF_night [n.u.]	32.3	38.3	0.041
TP_day [ms^2^]	2.747	3.248	0.152
TP_night [ms^2^]	2.923	3.174	0.616

DBP: diastolic blood pressure; HF: power in the high frequency range; HR: heart rate; LF: power in the low frequency range; n.u. : normalized units; pNN50: percentage of NN50; rMSSD: square root of the mean of the sum of the squares of differences between adjacent NN intervals; SBP: systolic blood pressure; SDNN: standard deviation of the average of NN intervals; TP: total power of variance of all NN intervals.

**Table 3:** Comparison of HRV parameters before and after 12-month treatment, in patients with HTN stratified by concomitant MetS.

	MetS[+], *n* = 70 Mean ± SD Before	MetS[+], *n* = 70 Mean ± SD After	Difference (MetS[+])	*p* value	MetS[-], *n* = 48 Mean ± SD Before	MetS[-], *n* = 48 Mean ± SD After	Difference (MetS[-])	*p* value
HR [L/min]	74.1	69.1	-5.0	0.023	72.3	66.5	-5.8	0.007
OSBP [mmHg]	142.1	120.1	-22.0	<0.0001	139.5	121.2	-18.3	<0.0001
ODBP [mmHg]	90.5	77.2	-13.3	<0.0001	89.3	77.6	-11.7	<0.0001
SDNN_24h [ms]	135.5	140.2	4.7	0.012	149.1	146.3	-2.8	0.665
SDNN_day [ms]	109.8	114.6	5.1	0.042	121.5	123.2	1.7	0.312
SDNN_night [ms]	90.5	95.2	4.7	0.189	94.6	96.7	2.1	0.885
rMSSD_24h [ms]	31.5	34.7	3.2	0.003	38.2	39.4	1.2	0.470
rMSSD_day [ms]	27.3	29.7	2.4	0.001	32.7	35.0	2.3	0.855
rMSSD_night [ms]	40.0	43.8	3.8	0.042	49.0	48.6	-0.4	0.112
pNN50_24h [%]	6.80	8.03	1.23	0.0002	10.79	11.42	0.69	0.665
pNN50_day [%]	4.66	5.56	0.90	0.001	7.63	8.91	1.28	0.885
pNN50_night [%]	12.65	14.29	1.64	0.051	19.28	18.13	-1.15	0.105
LF/HF_day [-]	4.50	4.02	-0.48	0.082	3.88	3.64	-0.24	0.136
LF/HF_night [-]	3.04	2.41	-0.63	0.550	2.01	1.88	-0.13	0.470
LF_day [n.u.]	70.5	67.9	-2.6	0.457	70.0	66.1	-3.9	0.136
LF_night [n.u.]	62.3	59.9	-2.4	0.403	55.8	53.7	-2.1	0.470
HF_day [n.u.]	23.0	26.1	3.1	0.082	24.1	28.4	4.3	0.074
HF_night [n.u.]	32.3	34.6	2.3	0.189	38.3	40.8	2.5	0.470
TP_day [ms^2^]	2.747	3.803	1.056	0.026	3.248	4.054	806	0.307
TP_night [ms^2^]	2.923	3.483	560	0.402	3.174	3.377	203	0.665

ODBP: office diastolic blood pressure; HF: power in the high frequency range; HR: heart rate; LF: power in the low frequency range; n.u.: normalized units; pNN50: percentage of NN50; rMSSD: square root of the mean of the sum of the squares of differences between adjacent NN intervals; OSBP: office systolic blood pressure; SDNN: standard deviation of the average of NN intervals; TP: total power of variance of all NN intervals.

## Data Availability

The datasets used and analysed during the current study are available from the corresponding author on reasonable request.

## Supplementary Materials

Supplementary Materials Supplementary Table 1. The correlations between changes (delta) in BP and HRV parameters after 12-month treatment.
