# Supplementary material for: Heart Rate Variability in Patients with Hypertension: the Effect of Metabolic Syndrome and Antihypertensive Treatment
Source: Cardiovasc Ther. 2020 Oct 13;2020:8563135. doi: 10.1155/2020/8563135 (PMC7582062; doi:10.1155/2020/8563135)

Supplementary Table 1. The correlations between changes (delta) in BP and HRV parameters after 12-month treatment.

|  | **MetS [+]** | | **MetS [+]** | |
| --- | --- | --- | --- | --- |
| delta | delta OSBP (R Spearman) | delta ODBP (R Spearman) | delta OSBP  (R Spearman) | delta ODBP (R Spearman) |
| SDNN_24h | -0.26 | -0.37* | -0.24 | -0.34* |
| SDNN_day | -0.32* | -0.36* | -0.16 | -0.28* |
| SDNN_night | -0.10 | -0.40* | -0.05 | -0.36* |
| rMSSD_24h | -0.31* | -0.47* | -0.14 | -0.36* |
| rMSSD_day | -0.28 | -0.49* | -0.13 | -0.32* |
| rMSSD_night | -0.20 | -0.35* | -0.12 | -0.40* |
| pNN50_24h | -0.29* | -0.49* | -0.11 | -0.34* |
| pNN50_day | -0.30* | -0.45* | -0.06 | -0.38* |
| pNN50_night | -0.32* | -0.41* | -0.10 | -0.08 |
| LF/HF_day | 0.06 | 0.04 | -0.18 | 0.11 |
| LF/HF_night | 0.27 | 0.08 | 0.04 | -0.08 |
| LF_day | -0.07 | -0.01 | -0.12 | 0.20 |
| LF_night | 0.20 | 0.10 | 0.16 | 0.04 |
| HF_day | -0.10 | -0.07 | 0.11 | -0.21 |
| HF_night | -0.22 | -0.10 | -0.17 | -0.18 |
| TP_day | -0.01 | -0.04 | -0.04 | -0.16 |
| TP_night | 0.07 | -0.11 | -0.05 | -0.34* |
| **p<0.05*  *ODBP –office diastolic blood pressure; HF – power in the high frequency range. LF – power in the low frequency range; n.u. – normalized units; pNN50 – percentage of NN50; rMSSD – square root of the mean of the sum of the squares of differences between adjacent NN intervals; OSBP – office systolic blood pressure; SDNN- standard deviation of the average of NN intervals; TP – total power of variance of all NN intervals* | | | | |


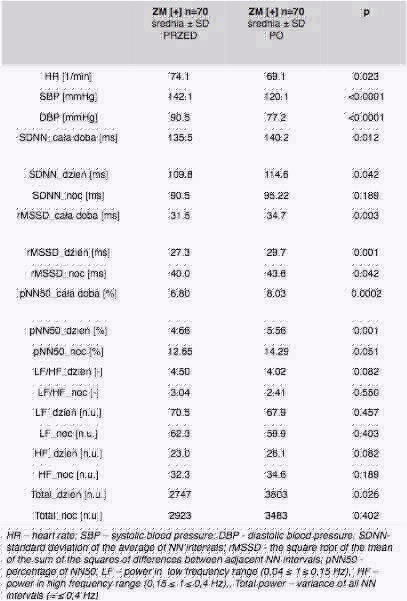

Supplement: Supplementary Materials — Supplementary Table 1. The correlations between changes (delta) in BP and HRV parameters after 12-month treatment. [file 8563135.f1.docx]
